# Foliar application of sodium selenite affects the growth, antioxidant system, and fruit quality of strawberry

**DOI:** 10.3389/fpls.2024.1449157

**Published:** 2024-08-12

**Authors:** Yuanxiu Lin, Shuaipeng Cao, Xiao Wang, Yin Liu, Ziqing Sun, Yunting Zhang, Mengyao Li, Yan Wang, Wen He, Yong Zhang, Qing Chen, Xiaorong Wang, Ya Luo, Haoru Tang

**Affiliations:** College of Horticulture, Sichuan Agricultural University, Chengdu, China

**Keywords:** strawberry, selenium, fruit quality, antioxidant system, plant growth

## Abstract

**Introduction:**

Selenium (Se) plays a vital role in various physiological processes in plants and is regarded as an essential micronutrient for human health as well.

**Methods:**

In this study, sodium selenite solution at 10, 40, 70, and 100 mg·L^-1^ concentrations was foliar sprayed, and the strawberry plant growth, antioxidant system, and fruit quality with an emphasis on sugar and acid content were assessed.

**Results:**

The results showed that 10 mg·L^-1^ of sodium selenite treatment promoted plant growth, while all the treated concentrations could enhance photosynthesis, the antioxidant system in leaves, the content of Se, and ascorbic acid in fruits. More importantly, 40 mg·L^-1^ sodium selenite treatment significantly increased fruit weight, total soluble solid, total phenolic content, and anthocyanins, as well as improved the shape index. Furthermore, it decreased the total flavonoid and proanthocyanidin content. Particularly, sodium selenite treatment at 40 and 70 mg·L^-1^ largely increased the ratio of soluble sugars to titratable acid. The changes of predominant sugars and organic acids during fruit development were further investigated. The sucrose, fructose, and glucose content was upregulated by sodium selenite treatment through upregulating the activities of sucrose phosphate synthase (SPS) and acid invertase, as well as the *FaSPS* expression. In addition, sodium selenite treatment inhibited the activity of citrate synthase and phosphoenolpyruvate carboxylase, rather than modulating their transcript levels to reduce the citric acid content.

**Conclusions:**

This work presented a potentially efficient approach to enhance plant growth and fruit quality and supplement Se in strawberry, providing insights into the mechanism of regulating sugar and acid metabolism by Se.

## 1 Introduction

Strawberry (*Fragaria × ananassa* Duch.) is widely popular worldwide due to its unique flavor, rich nutrition, and its economic importance. It has been suggested that every 100 g strawberry contains approximately 60 mg of ascorbic acid (AsA), 150 mg of potassium, 15 mg of calcium, and 100 mg of phenolic compounds such as proanthocyanidins and anthocyanins (Huma Qureshi et al., 2023; Salazar-Orbea et al., 2023). Therefore, strawberry has been considered a predominant health-beneficial dietary resource in the fresh market. In 2022, the global cultivation area of strawberry was 398 thousand hectares (ha), the yield totaled 24.1 million g/ha, and the harvest totaled 95.7 million tons (https://www.fao.org/faostat/en/#data/QCL). In China, the largest producer of strawberry, the production of strawberries was estimated at 33.6 tons, accounting for 35% of the total (https://www.fao.org/faostat/en/#data/QCL). However, the yield, quality, and phytochemical contents of fruit are often affected by various environmental factors (i.e. light and temperature) (Cervantes et al., 2019; Cui et al., 2021; Lauria et al., 2023) and cultural and management techniques (Tyagi et al., 2017). Foliar spraying of plant growth regulator or phytohormones is a simple way to improve fruit quality, but may raise food safety risks (Dias, 2019). Therefore, it is particularly important to develop a safe and environmentally friendly treatment method to resist the adverse environmental factors of strawberry production and improve the quality of strawberry fruit.

Selenium (Se) is a beneficial element that plays important roles in regulating plant growth and development, resistance to stress, and accumulation of secondary metabolites. Previous studies have demonstrated the significant role of Se in the regulation of growth in rice (Li et al., 2022), wheat (Lara et al., 2019; Sadak and Bakhoum, 2022), and sugarcane (De Araujo et al., 2023). It enhances plant nitrogen metabolism capacity by activating key enzymes, thereby improving plant chlorophyll synthesis and photosynthetic efficiency (Shahid et al., 2019; Da Silva et al., 2023). Moreover, the antioxidant system can effectively remove reactive oxygen species (ROS) in plants, which are enzymatic systems, including superoxide dismutase (SOD), catalase (CAT), and peroxidase (POD). Exogenous Se application has been shown to decrease oxidative stress in plants by upregulating antioxidative enzymes and improving the antioxidant system (Xu et al., 2024). Furthermore, an increasing number of studies have indicated that Se can effectively enhance AsA, soluble sugars, and anthocyanins content (Ryant et al., 2020; Wen, 2021), thereby improving the fruit quality of horticultural crops (Zahedi et al., 2019b; Liu et al., 2022). Recent research has also demonstrated that selenoproteins have strong antioxidant effects on plant metabolism through the regulation of the glutathione peroxidase (GSH) pathway (Zahedi et al., 2019a; Lanza and Reis, 2021). Moreover, Se enhances the activity of enzymes and non-enzyme compounds (such as AsA, flavonoids, and phenols), involved in clearing ROS and cell detoxification (Zahedi et al., 2019a; Lanza and Reis, 2021). In addition to its benefits for plants, Se is also essential for human health. Proper supplementation of Se can not only prevent various diseases but also help enhance immunity (Yang et al., 2022). According to the recommendations of the World Health Organization (WHO), adults need to supplement at least 55 μg of Se per day (Kipp et al., 2015). However, a quarter of the world’s population still falls below the normal standard (Tran et al., 2023). China has been reported as one of the 40 Se-deficient countries in the world, and there are approximately 39%-61% of the Chinese population has a daily Se intake of only around 30 μg (Dinh et al., 2018). Making dietary fruits rich in Se is becoming the safest and most efficient way for people to supplement Se.

Soil and foliar applications of inorganic Se constitute the main agronomic approaches for Se biofortification (Ros et al., 2016). Compared to soil application, foliar application is more effective as it circumvents the extensive fixation of selenite in the soil (Zawislanski et al., 2003). In recent years, different forms of Se have been increasingly used to increase Se concentration in food crops, vegetables, and fruits by foliar spraying, with the effect of improving yield and quality. A large number of studies have suggested that foliar application of Se can greatly improve plant growth, antioxidant activities, nutritional quality, and flavor of fruits (Lu et al., 2022; Wang et al., 2022). However, little is known about the effects of foliar spraying of Se on the Se biofortification and quality of strawberry fruit, especially the sugar and acid metabolism and underlying mechanism. This study investigated the influences of exogenous Se on the growth, antioxidant capacity, and phytochemical content, as well as the pivotal enzyme activities and corresponding gene expression levels during different developmental stages in the sugar and organic acid metabolism, and aimed to provide an insight into the quality regulatory mechanism in strawberry by Se and provide an alternative way to produce strawberry fruit with Se biofortification.

## 2 Materials and methods

### 2.1 Plant materials and treatments

Strawberry (*Fragaria × ananassa*, ‘Benihoppe’) seedlings were purchased from the strawberry planting base located in Deyang City, Sichuan Province. They were cultivated in pots with a substrate composed of nutrient soil, pastoral soil, perlite, and vermiculite in a 4:2:1:1 ratio, and placed in the experimental greenhouse of Sichuan Agricultural University. One plant per pot and a total of 250 pots were cultured. Conventional water and fertilizer management was carried out during the cultivation period. Subsequently, they were randomly divided into five groups with uniform growth. Starting from the budding stage (when 50% of the strawberries start budding) of strawberries, each group of strawberry seedlings was sprayed through the leaf surface with a Na_2_SeO_3_ solution with concentrations of 10, 40, 70, and 100 mg·L^-1^. The spray was applied once every 5 days until the leaves were dripping with water beads. A total of six sprays were conducted, with distilled water as the control. The photosynthetic parameters of strawberry seedlings were determined on the 15^th^ day after the first treatment. Ripened fruits were collected for quality indicator determinations, and fruits at big green (BG), white (W), partial red (PR), and full red (FR) stages were collected respectively according to our previous standard (Yue et al., 2022) for sugar and acid content and corresponding gene expression analysis. The samples were taken in the morning and immediately frozen with liquid nitrogen and stored in -80°C for further use. A total of 10 fruits were mixed as one biological replicate, and 3 biological replicates were determined for each indicant.

### 2.2 Determination of general plant growth and fruit quality indicators

Fresh samples were used for the determination of growth and photosynthesis indicators. Specifically, an electronic vernier caliper and a ruler were employed to measure the short stem thickness and plant height, respectively. The strawberry leaves were spread out and placed on a white paper with a ruler for photographing, and then the leaf area was measured by using Image J software (v.1.53).

The single fruit weight and the fruit transverse/vertical diameter were determined using fresh fruit samples by electronic scale and Vernier caliper (0.02 mm), respectively. The fruit shape index was calculated as the ratio of transverse to vertical diameter. Total soluble solids content (SSC) was detected in fresh juice using a refractometer (AtagoRx 5000, Atago Co.Ltd., Japan) and expressed as a percentage. The determination of total soluble sugars was performed using the modified anthrone colorimetric method (Jiang et al., 2023a). Briefly, approximately 0.1 g of frozen fruit sample was homogenated in 1 mL distilled water. The mixture was then diluted into 2% (w/v) anthrone-ethyl acetate and concentrated sulfuric acid, and subsequently boiled for 1 min. Eventually, the mixture was subject to a spectrophotometer to record the absorbance at 620 nm. An external standard was employed to quantify the soluble sugar content. Titratable acidity (TA) was determined by titration aliquots of fruit extract with 0.1 N NaOH to an end point of pH 8.2 and expressed as a percentage of citric acid. Determination of the AsA content was carried out according to the previously reported approach (Jiang et al., 2023a). The photographic density of fruit extract was read at 534 nm, and the result was represented as g of AsA per kg of fresh weight (FW).

### 2.3 Determination of chlorophyll content and photosynthetic parameters

Slightly modified from the previous method (Khan et al., 2023), 0.5 g of fresh strawberry leaves were accurately weighed and homogenized in 80% acetone solution. After reaction in the dark for 24 hours until the leaves turned completely white, the mixture was used for chlorophyll content measurement. We used 80% acetone as a blank and measured the absorbance value at 663 nm and 645 nm using a spectrophotometer (Multiskan GO, Thermo Co.Ltd., US) to calculate the content of Chl a and Chl b. The third functional leaf from the top of the strawberry plant was used for measuring net photosynthetic rate (Pn), stomatal conductivity (Gs), intercellular carbon dioxide (Ci), and transpiration rate (Tr) using a photosynthesometer (LI-6400, LI-COR Co.Ltd., US).

### 2.4 Determination of antioxidant enzyme activities

The activity of SOD was determined using the nitro blue tetrazolium (NBT) photoreduction method (Zhang et al., 2024). Specifically, 0.1 g of frozen leaf sample was weighed and homogenized in 1 mL extract solution containing 100 mM (pH7.0) phosphate buffer, and 1 mM EDTA. Followed by centrifugation at 10,000 g for 10 min, the supernatant was used for SOD activity measurement. One unit of SOD activity was defined as the amount of enzyme that caused a 50% inhibition of NBT reduction. The POD activity was measured using the guaiacol method (Liang et al., 2020). The working reagent consisted of 100 mL of 0.1 M phosphoric acid buffer with a pH of 6.0, 56 μL of guaiacol, and 38 mL of 30% H_2_O_2_. A total of 0.1 g of frozen leaves were weighed and homogenized with 1 mL of 20 mM KH_2_PO_4_. The absorbance at 470 nm was immediately measured; the absorbance readings were taken every minute for a continuous period of 5 minutes. It was determined that an absorbance change of 0.01 at 470 nm per minute corresponded to one unit of enzyme activity. For CAT activity measurement, 0.1 g of frozen sample was weighed and homogenized in 1.6 mL 50 mM pre-cooled phosphate buffer (pH 7.8). The change in the OD_240_ value within 5 minutes was measured, and it was determined that a decrease in the OD value by 0.01 per minute corresponded to an enzyme activity unit (Zhang et al., 2024).

### 2.5 Analysis of total flavonoid, phenolic, anthocyanin, and proanthocyanidin content

Total flavonoid content (TFC) and total phenolic content (TPC) were measured by using the previously described methods (Zhang et al., 2022). Specifically, 0.5 g of frozen fruit sample was weighed and mixed with 80% acetone and then incubated at room temperature for 1 hour. Subsequently, the clarified solution was collected by centrifuge to detect the content of TFC and TPC. The TFC was determined by the aluminum chloride colorimetric method, with quercetin as the reference substance. A calibration curve was made, and the results were expressed in mg of quercetin equivalent per kg of FW. The Folin Ciocalteu method was used to quantify the TPC, and the absorbance value of the solution was measured at 650 nm. Furthermore, 80% acetone was used as the blank, and gallic acid was used as the external standard to make a calibration curve. The result was expressed as the equivalent g of gallic acid per kg of sample.

The total anthocyanin content was detected by using an improved pH differential method (Zhang et al., 2018). Approaching 1 g of frozen fruit sample was extracted with solution consisting of acetic acid, water, acetone, and methanol in a ratio of 1:2:4:4 for 30 min. After a 4 hours of incubation in a water bath at 40 °C, the extract was diluted with 0.025 M KCl (pH 1.0) and 0.4 M NaAc (pH 4.5) and incubated at room temperature for 15 min. The absorbance was measured at 496 and 700 nm, and the content was expressed as the g of pelargonidin 3-glucoside per kg of FW. The developed 4-dimethylaminocinnamaldehyde (DMAC) method was conducted to analyze the proanthocyanidin content (Zhang et al., 2018). In a brief, 1.5 g of frozen fruit was extracted in a mix of acetone, water, and glacial acetic acid solution with a 150: 49: 1 ratio. The strawberry extract was stained with 0.1% DMAC at room temperature for approximately 15 min. Afterward, the absorbance value was measured at a wavelength of 640 nm. Procyanidin B2 was employed as the standard, and the total proanthocyanidin level was expressed as g of procyanidin B2 per kg of FW.

### 2.6 Malondialdehyde, free proline, and total antioxidant activity

Malondialdehyde (MDA) was assayed using a previously described procedure (Jiang et al., 2023a). A total of 0.5 g of frozen sample was completely homogenized with 10% trichloroacetic acid. The clear solution was blended with 0.67% 2-thiobarbituric and then boiled for 10 min and immediately cooled on ice. Finally, the absorption value at 450 nm, 523 nm, and 600 nm was recorded, and the result was represented as μmol per g FW. Acid ninhydrin colorimetry was employed to determine the free proline content in the treated strawberry leaf samples (Liang et al., 2020). Initially, 0.2 g of frozen sample was weighed and then mixed with 5 mL of 3% sulfosalicylic acid. The mixture was ground to a homogenate state on ice and was placed in a boiling water bath for 15 min with continuous hand shaking during this process. After extraction, it was cooled and centrifuged at 10,000 g for another 15 min to obtain the supernatant as the free proline extract. The absorbance value of the sample reaction liquid at 520 nm was immediately determined. The content of free proline in each sample could be calculated accurately according to its absorbance value measurement at the 520 nm wavelength.

The total antioxidant activities were estimated by FRAP (Ferric reducing antioxidant power) and DPPH (2,2-diphenyl-1-picrylhydrazyl) assays. According to the previous approaches (Jiang et al., 2023a), 1g of frozen sample was extracted with 10 mL of ethanol (ground thoroughly in a mortar, then sonicated for 10 min). After centrifuging for 15 min, 0.2 mL of sample solution was added to 2.8 mL of 60 μM DPPH, and then the mixture was allowed to stand in the dark for 30 min at room temperature. The optical density of the mixture at 517 nm was read. The clearance activity was evaluated based on the percentage inhibition of DPPH. For FRAP measurement (Zhang et al., 2022), 20 μL of the sample extract was thoroughly mixed with 1.8 mL of fresh working FRAP reagent (300 mM pH 3.6 acetate buffer, 10 mM TPTZ, and 20 mM FeCl_3_ · 6H_2_O in a volume ratio of 10:1:1). The mixture was incubated at 37 °C for 30 min, and the absorption value at 593 nm was recorded. The clearance effect was expressed in mmol kg^-1^ FW.

### 2.7 Determination of glucose, fructose, sucrose, citric acid, and malic acid contents

The concentration of predominant soluble sugar (glucose, fructose, and sucrose) and organic acids (citric acid, malic acid) was determined by the HPLC method (Zhang et al., 2023). Briefly, 0.5 g of frozen sample was added to 5 mL of distilled water. After sonicating for 30 min, the mixture was centrifuged at room temperature for 30 min. Subsequently, 1 mL of the supernatant was filtered through a 0.45 μm filter membrane, and 10 μL of which was injected into HPLC system for substantial determination of sugar and organic acid content. Sucrose, fructose, and glucose was detected using a refractive index detector, and separated with an NH2-RP chromatographic column (4.6 mm × 250 mm, 5 μm, Agela Technologies, Shanghai, China). Acetonitrile/water (75:25, v/v) was used as the mobile phase. The column temperature was kept at 25 °C, and the flow rate was set as 1 mL min^-1^. Citric acid and malic acid were detected using a diode array detector (DAD) detector, and separated by using a C18-WP (4.6 × 250 mm, 5 μm) chromatographic column. The mobile phase was methanol in 0.2% phosphoric acid (3:97, v/v), the detection wavelength was 210 nm, the flow rate was 0.8 mL min^-1^, and the column temperature was kept at 40°C.

### 2.8 Measurement of enzyme activities

The sugar metabolism-related enzymes were extracted according to a previous report (Yu et al., 2017). Briefly, 1.0 g of frozen fruit tissue was homogenized on ice with 5.0 mL of precooled 0.1 M phosphate buffer (pH 7.5, 5.0 mM MgCl_2_, 1.0 mM EDTA, 2.5 mM DTT, and 0.1% (v/v) Triton X-100). The activities of sugar metabolism related enzymes including acid invertase (AI), neutral invertase (NI), sucrose phosphate synthase (SPS), sucrose synthase synthesis (SS-s) and the sucrose synthase cleavage (SS-c), as well as the activities of citrate synthase (CS), phosphoenol pyruvate carboxylase (PEPC) and isocitrate dehydrogenase (IDH) involved in organic acid metabolism were detected and evaluated using different reaction solutions according to the method of Gao et al (Gao et al., 2022).

### 2.9 Quantitative RT-PCR analysis

Quantitative RT-PCR (qRT*-*PCR) analysis were performed on a CFX96 RT-qPCR system (Bio-Rad, USA) by using SYBR Green Premix Ex Taq™ kit (Takara, Japan). An improved cetyltrimethylammonium bromide (CTAB) method was used to isolate the total RNA, and the PrimeScript™ RT reagent kit with gDNA Eraser (Takara, Japan) was used to synthesis the first strand cDNA. A total of 10 μL reaction for each gene was carried out with specific primers, which were designed using NCBI online tool, and listed in **Supplementary Table S1**. The relative expression of genes to the internal reference (Jiang et al., 2023b) was calculated using the 2^-ΔΔCt^ method (Livak and Schmittgen, 2001).

### 2.10 Statistical analysis

All experiments were repeated three times with three independent biological replicates. All the present data were statistically analyzed using Prism software (version 9.5.0). Experimental data were expressed as mean values of three biological replicates ± standard deviation (SD). The LSD multiple comparisons test was used to compare the differences between the control and treatment. Results with a P value below 0.05 and 0.01 were considered as statistically significantly different.

## 3 Results

### 3.1 Sodium selenite treatments affected plant growth

It was observed that the application of low concentrations (10 and 40 mg·L^-1^) of Na_2_SeO_3_ significantly enhanced plant growth. These resulted in a 19.1% and 7.1% increase in plant height, and a 12.7% and 5.0% increase in stem thickness, respectively. Additionally, leaf area increased by 77.7% and 47.1%. However, treatment with a high concentration of Na_2_SeO_3_ at 70 mg·L^-1^ did not lead to significant changes in stem thickness, plant height, or leaf area. Notably, the application of 100 mg·L^-1^ Na_2_SeO_3_ treatment had no significant impact on plant stem size but did result in a significant reduction in both plant height and leaf area (**Table 1**).

**Table 1:** The effect of Na_2_SeO_3_ treatment with different concentrations on plant growth.

Na_2_SeO_3_ concentration (mg·L^-1^)	Stem thickness (cm)	Plant height (cm)	Leaf area (cm^2^)
0	2.16 ± 0.15 bc	9.58 ± 1.28 bc	11.97 ± 1.59 c
10	2.44 ± 0.15 a	11.41 ± 2.09 a	21.26 ± 3.44 a
40	2.26 ± 0.17 ab	10.26 ± 1.82 ab	17.60 ± 2.43 b
70	1.99 ± 0.32 c	8.30 ± 0.90 cd	11.22 ± 1.36 cd
100	1.97 ± 0.21 c	7.72 ± 0.72 d	8.60 ± 1.69 d

The lower-case letters following the mean values indicate significant difference at P ≤ 0.05 level.

### 3.2 Sodium selenite treatments increased photosynthesis

**Figure 1:**
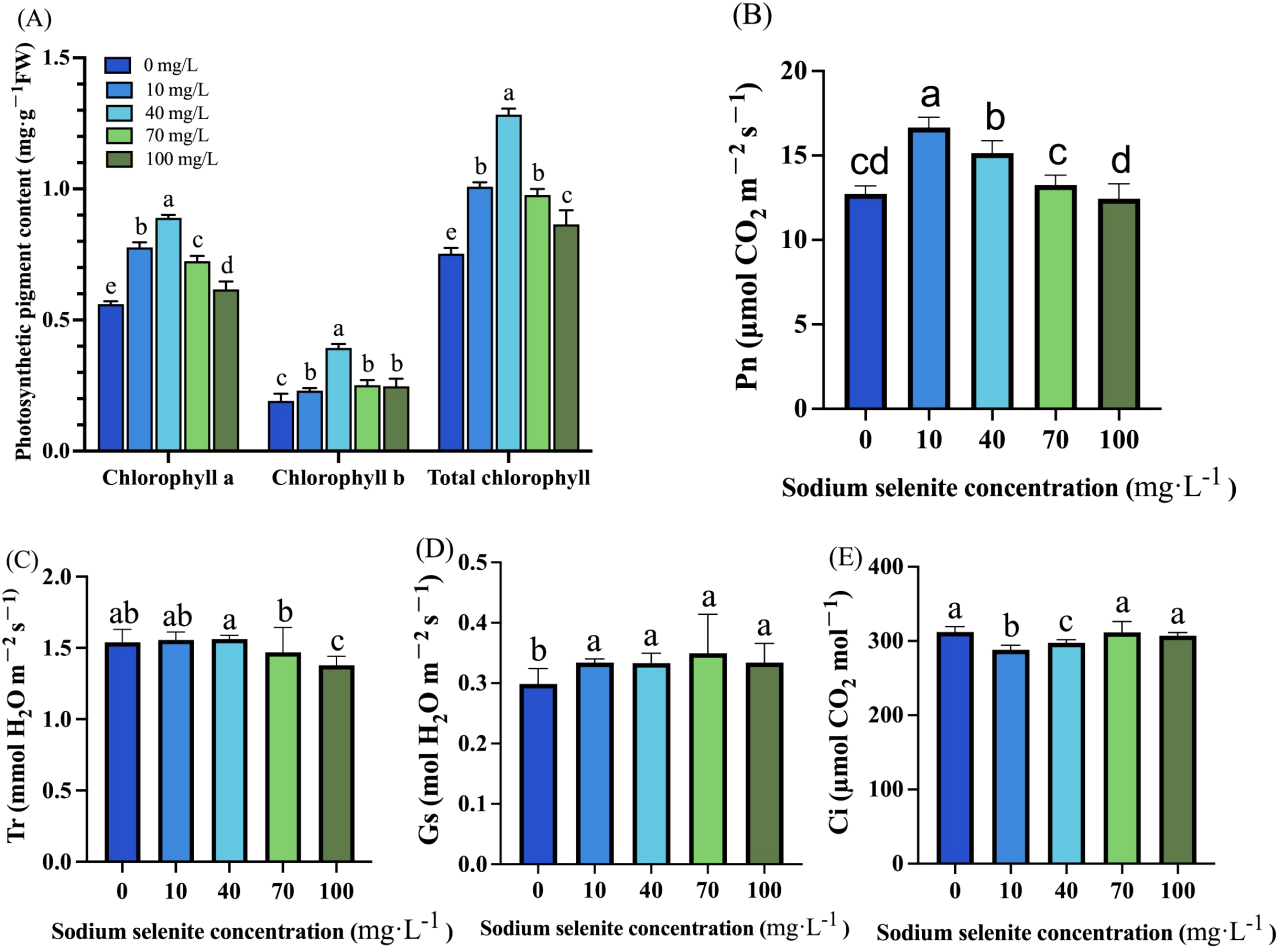
The effects of Se treatments on photosynthesis related parameters in strawberry plants. **(A)** Chlorophyll a, chlorophyll b, and total chlorophyll content in strawberry leaves. **(B)** Leaf area of strawberry under different concentrations of the Na_2_SeO_3_ treatment. **(C-E)** The Pn, Tr, Gs, and Ci changes in response to different Na_2_SeO_3_ treatments. Columns with error bars represent the mean values of three biological replicates ± standard deviation. The LSD multiple comparisons test was used to compare the differences between control and treatment. The lower-case letters indicate a significant difference at the *P ≤* 0.05 level.

We observed that treatments with various concentrations of Na_2_SeO_3_ significantly enhanced the levels of chlorophyll a, chlorophyll b, and total chlorophyll in strawberry leaves. Compared to the control group, the content of chlorophyll an increased significantly by 38.75%, 58.62%, 30.36%, and 10.71% under Na_2_SeO_3_ treatments at concentrations of 10, 40, 70, and 100 mg·L^-1^ respectively. Similar trends were observed for chlorophyll b and total chlorophyll content with the highest levels recorded under treatment with Na_2_SeO_3_ at a concentration of 40 mg·L^-1^ and the lowest levels under treatment with Na_2_SeO_3_ at a concentration of 100 mg·L^-1^ (**Figure 1A**). The Pn in the 10 mg·L^-1^ and 40 mg·L^-1^ treatment groups was significantly increased (30.69% and 18.92%, respectively), while the 70 mg·L^-1^ and 100 mg·L^-1^ treatments showed no obvious change in Pn (**Figure 1B**). The Tr of strawberry plants treated with low Na_2_SeO_3_ concentrations (10 mg·L^-1^ and 40 mg·L^-1^) displayed no significant alterations, while the high concentrations at 70 mg·L^-1^ and 100 mg·L^-1^ decreased Tr by 4.60% and 10.50%, respectively (**Figure 1C**). The Gs of strawberry plants significantly increased in all treatment groups, as depicted in **Figure 1D**. Ci was significantly decreased by the 10 mg·L^-1^ and 40 mg·L^-1^ treatments, while it was not changed by the 70 mg·L^-1^ and 100 mg·L^-1^ treatments (**Figure 1E**).

### 3.3 Effects of Se treatment on antioxidant characteristics in leaves

**Figure 2:**
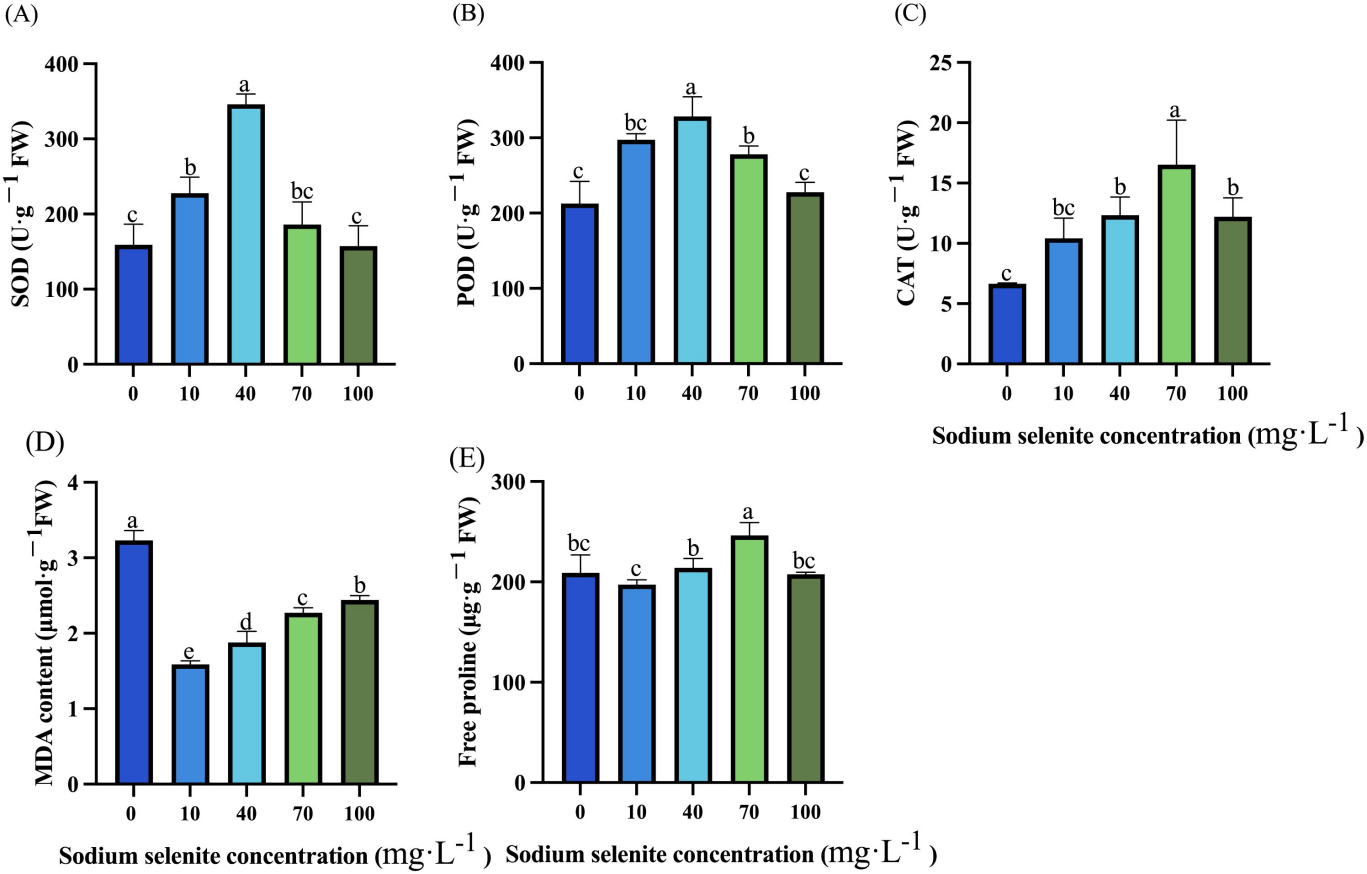
Effects of Na_2_SeO_3_ treatments on antioxidant characteristics in strawberry. **(A-C)** Changes in antioxidant enzymes SOD, POD, and CAT activities under treatment with different Na_2_SeO_3_ concentrations. **(D)** MDA content under treatments with different Na_2_SeO_3_ concentrations. **(E)** Free proline in strawberry leaves under Na_2_SeO_3_ treatments. Columns with error bars represent the mean values of three biological replicates ± standard deviation. The LSD multiple comparisons test was used to compare the differences between control and treatment. The lower-case letters indicate a significant difference at the *P ≤* 0.05 level.

Compared to the control group, treatments with 10 mg·L^-1^ and 40 mg·L^-1^ Na_2_SeO_3_ respectively resulted in a significant increase in SOD activity of 3.27% and 17.74% (**Figure 2A**). Except for the 100 mg·L^-1^ treatment, treatments with 10, 40, and 70 mg·L^-1^ Na_2_SeO_3_ significantly increased POD activity by 39.71%, 54.37%, and 30.64% respectively (**Figure 2B**). However, all treatment groups exhibited enhancement in CAT activity ranging from an increase of 56.90% to 148.62% compared to the control group; among them, the effect was not significant at a concentration of 10 mg·L^-1^, while the best effect was observed at a concentration of 70 mg·L^-1^ (**Figure 2C**). Moreover, all Se treatment groups significantly reduced the content of MDA, among which 10, 40, 70, and 100 mg·L^-1^ Na_2_SeO_3_ reduced 51.80%, 41.82%, 29.65%, and 24.47%, respectively (**Figure 2D**). Notably, Na_2_SeO_3_ treatments did not have a great impact on the accumulation of free proline, and only the 70 mg·L^-1^ Na_2_SeO_3_ treatment group significantly increased the content of free proline in strawberry leaves by 17.78% (**Figure 2E**).

### 3.4 Analysis of the Se accumulation

**Figure 3:**
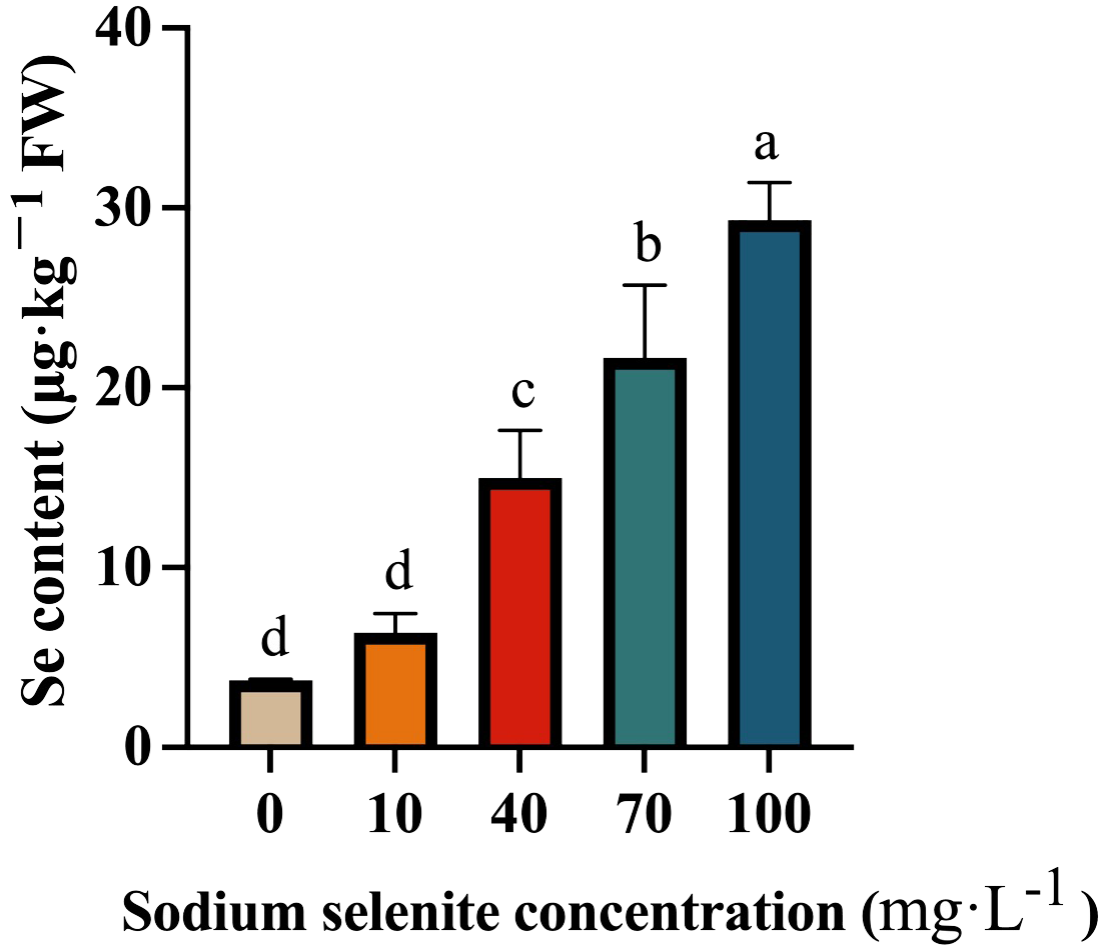
The Se content in strawberry treated with different Na_2_SeO_3_ concentrations. Columns with error bars represented the mean values of three biological replicates ± standard deviation. The LSD multiple comparisons test was used to compare the differences between control and treatment. The lower-case letters indicate a significant difference at the *P ≤* 0.05 level.

The Se content in strawberry fruit gradually increased as the Na_2_SeO_3_ concentration increased (**Figure 3**). In the control fruit, the Se content was relatively low, and it was slightly increased by the 10 mg·L^-1^ Na_2_SeO_3_ treatment, but no significant difference was observed. However, the Se content in the 40, 70, and 100 mg·L^-1^ Se-treated fruit was significantly higher than that in the control group (**Figure 3**).

### 3.5 Effects of Se on fruit weight and shape

As shown in **Table 1**, all the concentrations of Se treatments improved the single fruit weight. However, only the 70 mg·L^-1^ treatment showed a statistically difference between the Se treatment and control. Similarly, Se treatment increased the transverse and vertical of strawberry fruit regardless of the concentrations, with 70 mg·L^-1^ the most effective. No significant difference was observed between the control and Se-treated fruit except for 40 mg·L^-1^, which significantly increased the fruit shape index (**Table 2**). These results indicated that medium Se concentration (40 and 70 mg·L^-1^) may have positive effects on fruit weight and shape rather than low (10 mg·L^-1^) or high (100 mg·L^-1^) concentrations.

**Table 2:** The effect of Na_2_SeO_3_ treatment with different concentrations on fruit weight and shape.

Na_2_SeO_3_ concentration (mg·L^-1^)	Single fruit weight (g)	Transverse diameter (mm)	Vertical diameter (mm)	Shape index
0	12.97 ± 2.58 b	2.82 ± 0.26 b	3.91 ± 0.36 c	1.39 ± 0.11 b
10	14.1 ± 5.29 ab	3.00 ± 0.22 ab	4.32 ± 0.42 abc	1.44 ± 0.10 ab
40	13.16 ± 5.01 b	2.90 ± 0.42 ab	4.40 ± 0.39 ab	1.53 ± 0.11 a
70	16.99 ± 4.82 a	3.24 ± 0.42 a	4.64 ± 0.327 a	1.44 ± 0.12 ab
100	15.39 ± 4.99 ab	2.98 ± 0.32 ab	4.13 ± 0.38 bc	1.39 ± 0.07 b

The lower-case letters following the mean values indicate significant difference at P ≤ 0.05 level.

### 3.6 Effects of Se treatments on phytochemicals and antioxidant capacity

**Figure 4:**
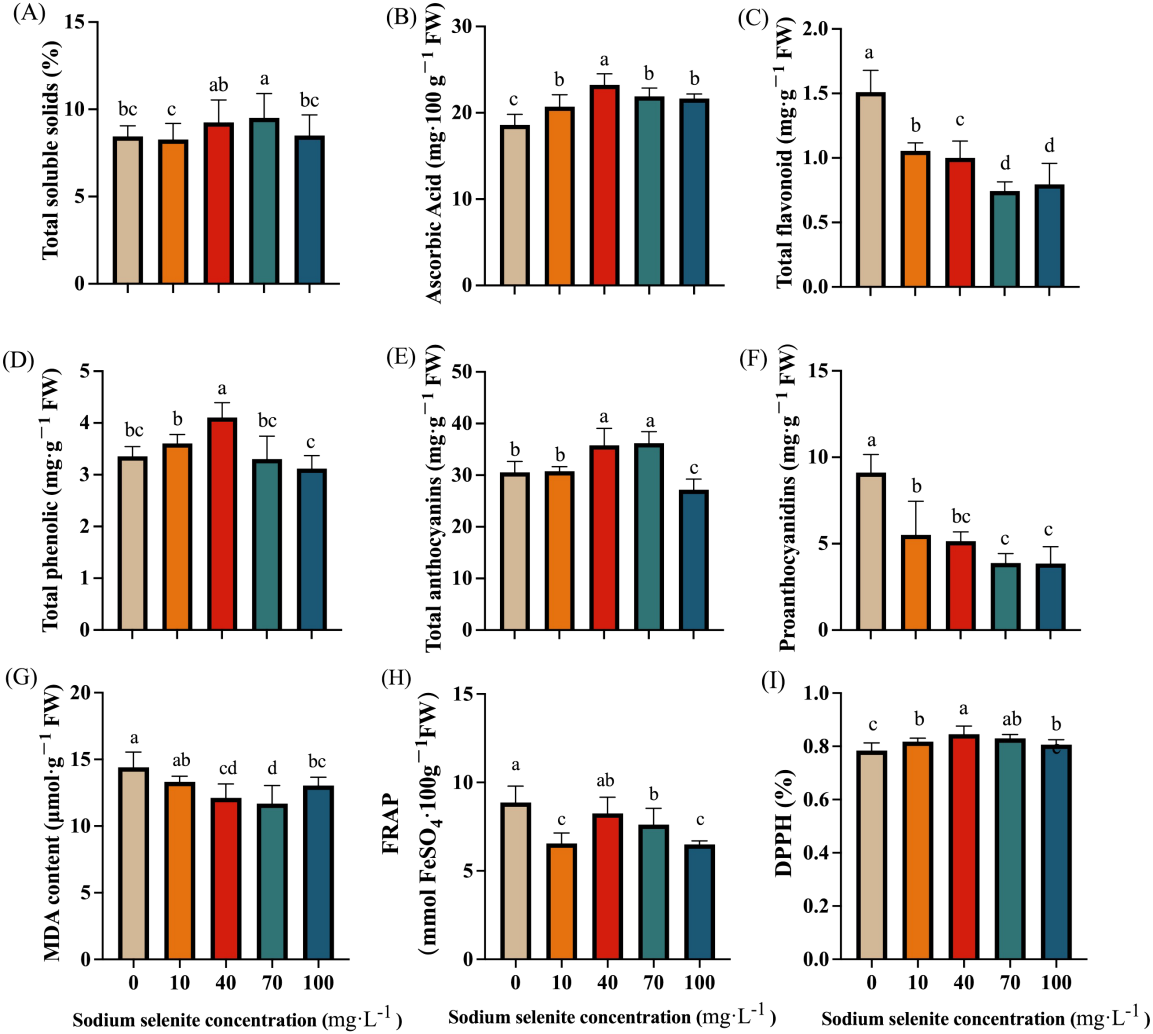
The effects of Se treatment on the content of phytochemicals and antioxidant capacity. **(A)**, Total soluble solids content; **(B)**, Ascorbic acid content; **(C)**, Total flavonoid content; **(D)**, Total phenolic content; **(E, F)**, Total anthocyanins and proanthocyanidins content; **(G)**, MDA content; **(H, I)**, FRAP and DPPH. Columns with error bars represent the mean values of three biological replicates ± standard deviation. The LSD multiple comparisons test was used to compare the differences between control and treatment. The lower-case letters indicate a significant difference at the *P ≤* 0.05 level.

Apart from 70 mg·L^-1^, the TSS was not significantly affected by the Se treatments (**Figure 4A**). In addition, the AsA content in strawberry fruits was significantly increased, with the 40 mg·L^-1^ treatment showing the best effect with a 24.81% increase compared to the control group (**Figure 4B**). By contrast, the total flavonoid content was significantly decreased by the Se treatments (**Figure 4C**) regardless of the concentrations. The fruit treated with 70 mg·L^-1^ sodium selenite had the lowest level. The Se treatments at 10 and 40 mg·L^-1^ increased the total phenolic content, while the 70 and 100 mg·L^-1^ Se concentrations had the opposite effect (**Figure 4D**). Additionally, the 40 and 70 mg·L^-1^ Se-treated fruit displayed a significant (P < 0.05) increase, while the 100 mg·L^-1^ Se-treated fruit exhibited a significant decrease in anthocyanin content (**Figure 4E**). There was no obvious difference between the 10 mg·L^-1^ Se treatment and control groups in terms of anthocyanin content (**Figure 4E**). The proanthocyanidin content was significantly decreased by the Se treatments regardless of concentration (**Figure 4F**).

Overall, the MDA content in strawberry fruits treated with exogenous Na_2_SeO_3_ decreased. However, the effect of a low concentration (10 mg·L^-1^) of Na_2_SeO_3_ was not significant. The MDA content in the 40, 70, and 100 mg·L^-1^ Na_2_SeO_3_ treatment groups was significantly decreased by 15.82%, 18.81%, and 9.43% respectively (**Figure 4G**). The FRAP values of all treatment groups showed a decreasing trend compared to the control group, with decreases of 26.10%, 7.02%, 14.62%, and 26.93% respectively. Only the difference for the 40 mg·L^-1^ treatment was not significant (**Figure 4H**). However, the DPPH value of strawberry fruit after exogenous Se treatment increased, with only the difference for the 100 mg·L^-1^ treatment not being significant (**Figure 4I**). The DPPH value for the 40 mg·L^-1^ treatment was significantly higher than that of both the control group and the other Na_2_SeO_3_ concentration treatments (**Figure 4I**).

### 3.7 The effects of Se treatments on sugar and acid content

**Figure 5:**
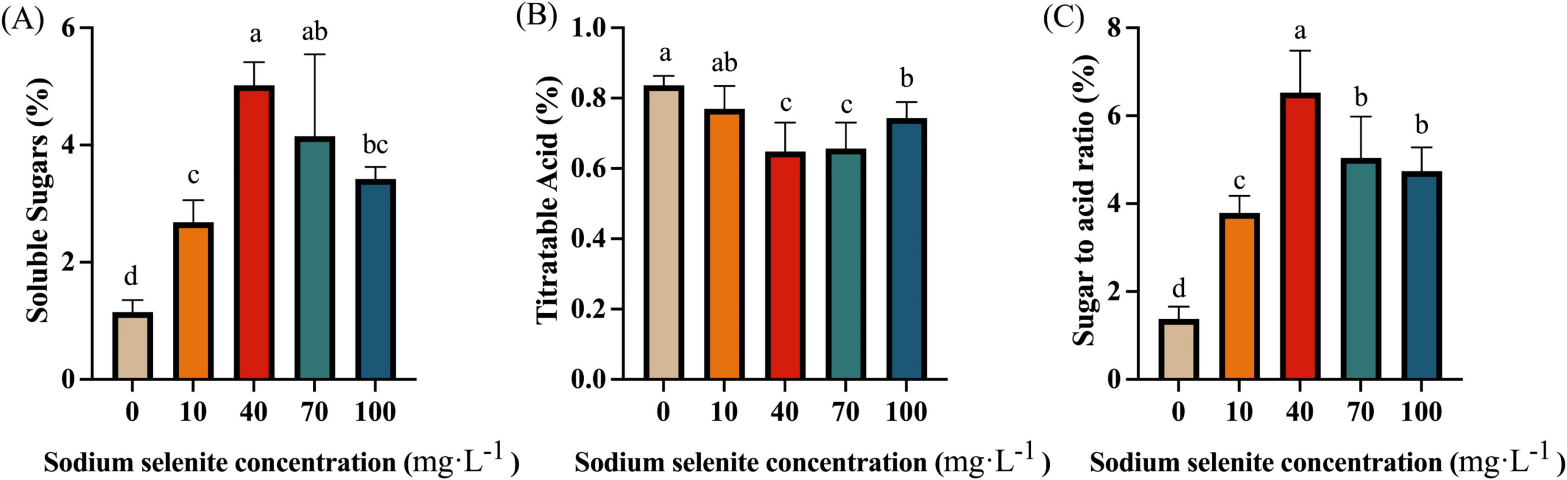
The effects of Se application on sugar and acid content. **(A)**, Soluble sugars content; **(B)**, Titratable acid content; **(C)**, The ratio of sugar to acid. Columns with error bars represent the mean values of three biological replicates ± standard deviation. The LSD multiple comparisons test was used to compare the differences between control and treatment. The lower-case letters indicate a significant difference at the *P ≤* 0.05 level.

Sugar and acid are one of the important factors affecting the flavor and quality of strawberry fruit. As shown in **Figure 5A**, regardless of the concentration of Na_2_SeO_3_ solution, the treatment group contained a notably greater level of soluble sugars than the control group. The highest soluble sugar level was detected in 40 mg·L^-1^ Se-treated fruit, followed by the 70, 100, and 10 mg·L^-1^ treatments (**Figure 5A**). In contrast, Se treatments effectively reduced the titratable acid content of strawberry fruit, with 40 and 70 mg·L^-1^ Na_2_SeO_3_ showing the most significant effects. There was no significant difference in TA content between the 10 mg·L^-1^ treatment and control fruit (**Figure 5B**). As shown in **Figure 5C**, the exogenous Se treatments substantially increased the ratio of sugar/TA (P < 0.01). Among them, 40 and 70 mg·L^-1^ Na_2_SeO_3_ had the most consequential effects, showing higher sugar to acid ratio than that of the 10 and 100 mg·L^-1^ treatments.

### 3.8 Effects of Se treatments on sugar and acid content during fruit development

**Figure 6:**
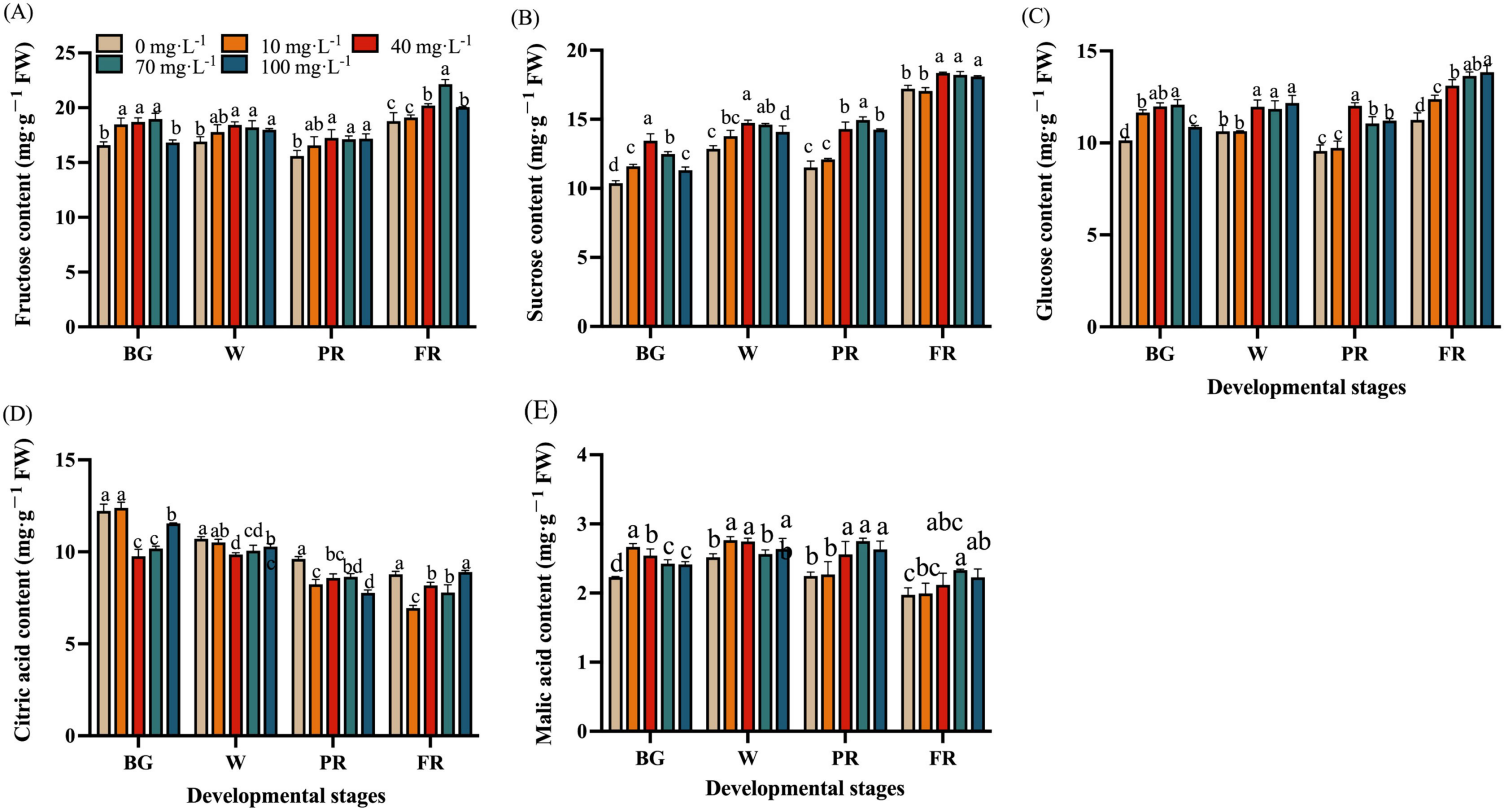
Effects of Se treatments on sugar and acid content during fruit development. **(A)**, Fructose content; **(B)**, Sucrose content; **(C)**, Glucose content; **(D)**, Citric acid; **(E)**, Malic acid content. Columns with error bars represent the mean values of three biological replicates ± standard deviation. The LSD multiple comparisons test was used to compare the differences between control and treatment. The lower-case letters indicate a significant difference at the *P ≤* 0.05 level.

To better understand the effects of Se treatments on sugar and acid metabolism in strawberry, the content of the main sugars (fructose, sucrose, and glucose) and organic acids (citric acid and malic acid) were measured during fruit development and ripening. The results showed the fructose and glucose content of strawberry fruits tended to stabilize from the BG stage, but the sucrose content gradually increased during all the developmental stages (**Figures 6A–C**). Under the treatment with 40 mg·L^-1^ and 70 mg·L^-1^ Na_2_SeO_3_ solutions, the content of fructose, sucrose, and glucose was significantly higher than that of the control group. Whereas the 100 mg·L^-1^ Se treatment showed no significant change in fructose content at the BG stage, while no significant changes in all the detected sugars during the developmental stages were found (**Figures 6A–C**).

Overall, the citric acid content was found gradually decreased with fruit ripening, Se treatment notably decreased the citric acid content in strawberry comparing to the control (**Figure 6D**). Interestingly, the citric acid content in the 40 and 70 mg·L^-1^ Na_2_SeO_s_ treatment groups was significantly lower than that in the other groups at the BG stage, indicating that these treatments could reduce the citric acid content in strawberry at the early developmental stage. However, it was found that malic acid content was relative stable during fruit developmental stages, Se treatment might increase the content of malic acid (**Figure 6E**).

### 3.9 Analysis of the enzyme activities and gene expression in sugar metabolism

**Figure 7:**
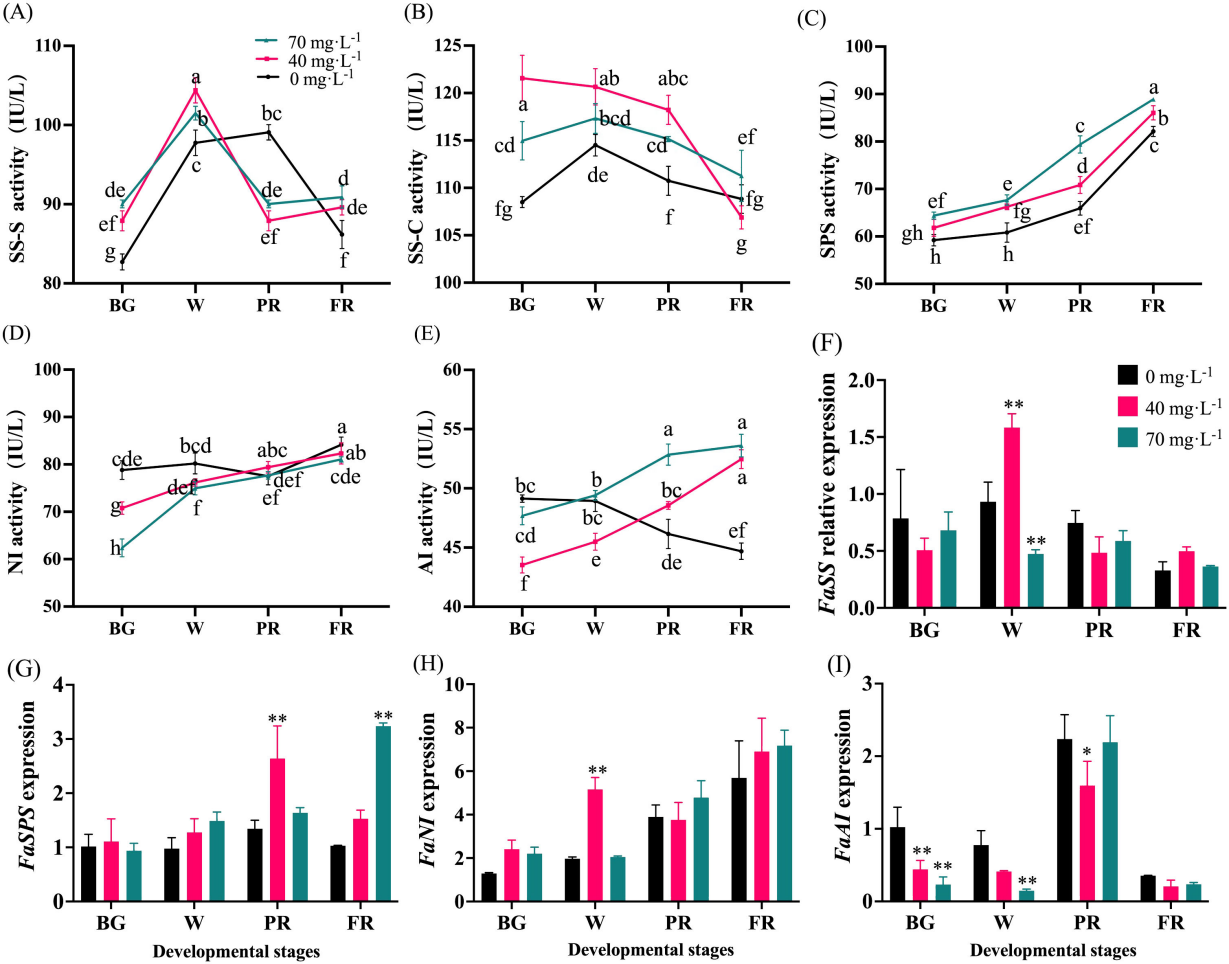
Changes of sugar metabolism-involved enzyme activities and gene expression during fruit development. **(A)**, Sucrose synthase synthesis activity; **(B)**, Sucrose synthase cleavage activity; **(C)**, Activity of sucrose phosphate synthase; **(D)**, Neutral invertase activity; **(E)**, Acid invertase activity; **(F)**, Sucrose synthase gene expression; **(G)**, Sucrose phosphate synthase gene expression; **(H)**, Neutral invertase gene expression; **(I)**, Acid invertase gene expression. BG, W, PR, and FR indicate fruit at the big green, white, partial red, and full red stages, respectively. Columns with error bars represent the mean values of three biological replicates ± standard deviation. The LSD multiple comparisons test was used to compare the differences between control and treatment. The lower-case letters indicate a significant difference at the *P ≤* 0.05 level.

Changes in the expression of critical genes involved in sugar metabolism, and the activities of corresponding enzymes during fruit development were analyzed in strawberry (**Figure 7**). As a result, SSs and SSc activities showed similar trends, which initially increased from the BG to W stage, and thereafter decreased (**Figures 7A, B**). During the early fruit development period, exogenous Se stimulated SSs and SSc activities. However, there was no significant difference (P > 0.05) in their activities between the Se treatment and control groups at the end of development (**Figures 7A, B**). An overall increase in activity was observed in SPS and NI for both Se-treated and control fruits (**Figures 7C, D**). During the entire developmental process, Se application, especially with a concentration of 70 mg·L^-1^, significantly enhanced SPS activity (**Figure 7C**) whereas lower NI activity was observed in fruit subjected to Se treatment during the early developmental stages (**Figure 7D**). No obvious change in NI activity between Se-treated and control fruit was found (**Figure 7D**). Interestingly, AI activity displayed an overall decreasing trend in control fruit and exhibited an opposite trend in Se-treated fruit (**Figure 7E**).

The transcript levels of the detected genes were not altered much by Se treatment. *FaSS* expression increased first and then decreased during the fruit development process, and no significant difference between control and Se-treated fruit was found (**Figure 7F**). Similar increasing trends of *FaSPS* and *FaNI* expression were found in control and Se-treated fruit (**Figures 7G, H**). The expression of *FaSPS* was at a higher level in 40 mg·L^-1^ and 70 mg·L^-1^ Se-treated fruit at the PR and FR stages, respectively (**Figure 7G**). In addition, fruits subjected to the 40 mg·L^-1^ Se treatment had a higher level of *FaNI* expression at the W stage (**Figure 7H**). However, no significant changes in *FaSPS* and *FaNI* expression were found in the Se-treated and control fruit. In general, Se treatment suppressed *FaAI* expression during fruit development (**Figure 7I**).

### 3.10 Analysis of the enzyme activities and gene expression in organic acid metabolism

**Figure 8:**
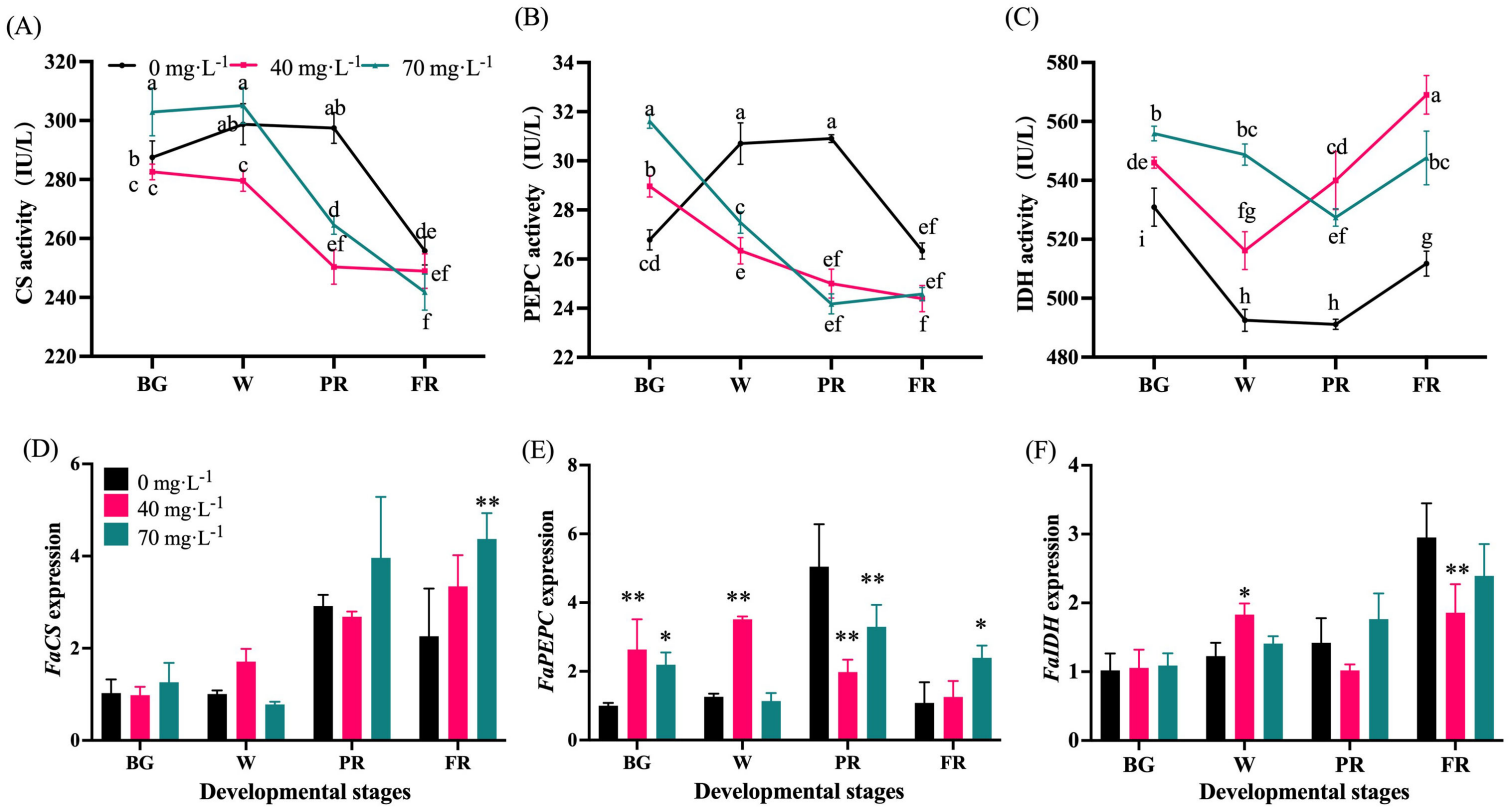
Enzyme activities and the transcript levels of acid metabolism-involved genes. **(A)**, Activity of citrate synthase; **(B)**, Phosphoenolpyruvate carboxylase activity; **(C)**, Isocitrate dehydrogenase activity; **(D)**, Citrate synthase expression; **(E)**, Phosphoenolpyruvate carboxylase expression; **(F)**, Isocitrate dehydrogenase expression. BG, W, PR, and FR indicate fruit at the big green, white, partial red, and full red stages, respectively. Columns with error bars represent the mean values of three biological replicates ± standard deviation. The LSD multiple comparisons test was used to compare the differences between control and treatment. The lower-case letters indicate a significant difference at the *P ≤* 0.05 level.

As shown in **Figure 8**, CS activity maintained at a relative stable high level during the early fruit development but sharply decreased from the W to FR stages in Se-treated fruits. While in control fruit, it stayed at a stable high level until the PR stage (**Figure 8A**). Compared to the control, the Se application repressed CS activity at the end of fruit development (**Figure 8A**). An initial increase and then decrease in PEPC activity was observed for the control group, while a gradual downward trend was found for the Se treatment group (**Figure 8B**). Furthermore, the Se treatment significantly inhibited the PEPC activity from the W to FR stage and no obvious difference was found for different concentrations (**Figure 8B**). Moreover, IDH activity showed a decreasing trend at the early developmental stages, with an increase at the end of development. It was greatly upregulated by Se treatment regardless of the concentrations (**Figure 8C**). The transcript levels of *FaCS* and *FaIDH* showed general upward trends (**Figures 8D, F**). Fruit treated with 70 mg·L^-1^ Se had the highest *FaCS* expression at the FR stage, showing a significant difference compared to other groups (**Figure 8D**). The 40 mg·L^-1^ Se treatment increased and reduced *FaIDH* expression at the W and FR stages respectively (**Figure 8F**). In addition, the Se-treated fruits showed a higher level of *FaPEPC* expression at the early developmental stage and a lower level at the end of development than those treated with the control (**Figure 8E**).

## 4 Discussion

It has been demonstrated in diverse species that Se can protect plants from the damage caused by abiotic stress and promote plant growth (Das et al., 2019; Hu et al., 2023). A previous study has reported that Se application with a low concentration had no obvious effects but a high concentration significantly decreased fresh weight in pea sprouts (Tan et al., 2022). Similarly, our results found that the plants treated with 10 mg·L^-1^ and 40 mg·L^-1^ Na_2_SeO_3_ were significantly taller and had larger stem diameters and greater leaf areas than those in the control group (**Table 1**). However, a high concentration (100 mg·L^-1^) had no obvious effect or even opposite influence on the plant height, stem diameters, and leaf areas (**Table 1**). These results indicated that an application of a low concentration of Se is beneficial to plant growth, while Se applied with a high concentration could inhibit plant growth (Cheng et al., 2024). Furthermore, exogenous Se could promote chlorophyll content, net photosynthetic rate, and stomatal conductance of grape leaves under salt stress and restore their photosynthetic capacity. It was found that a 30-90 mg·L^-1^ Se solution could promote the synthesis of chlorophyll b in the leaves of *Camelina Enshi*, thus promoting its photosynthetic efficiency (Lei and Wu, 2011). In this study, foliar application of Na_2_SeO_3_ with different concentrations increased the contents of chlorophyll a and b and enhanced the stomatal conductivities of strawberry plants. However, only the 10 mg·L^-1^ and 40 mg·L^-1^ treatments increased the net photosynthetic rate. This is consistent with previous studies, indicating that comparatively low concentrations of Na_2_SeO_3_ solution can enhance chlorophyll accumulation of plants, and thus enhance the intensity of photosynthesis. In addition, previous studies have shown that exogenous Se could enhance the activities of SOD, POD, and CAT enzymes in plants and reduce the content of MDA, a product of lipid peroxidation, thus alleviating plant damage caused by biological and abiotic stresses (Ashraf et al., 2018). Consistent with this, it was found in this study that foliar spraying of Na_2_SeO_3_ could increase the activities of SOD, POD, and CAT, but significant differences were found only in the groups treated with medium or low concentrations (10, 40, and 70 mg·L^-1^). In addition, Na_2_SeO_3_ treatment significantly reduced the content of MDA and greatly retarded the damage caused by lipid peroxidation to strawberries (**Figure 4**). However, Na_2_SeO_3_ treatment had no significant effect on the accumulation of free proline (**Figure 4**), which suggested that Na_2_SeO_3_ regulates stress resistance of strawberry plants mainly through antioxidant enzymes rather than amino acids.

Se is an essential trace element for human beings and insufficient intake of Se (lower than 55 μg per day for adults) can easily cause a decrease in immunity and thus threaten human health. However, over 50% of China’s soils are Se-deficient, resulting in low Se content in plant dietary sources and Se deficiency in approximately 39%-61% Chinese population. Therefore, Se enrichment has emerged as one of the concerns regarding the quality of food nutrition. Normally, a ripened strawberry fruit contains approximately 6 μg·kg^-1^ of Se (Lu et al., 2022). The cultivation solution addition or foliar application of inorganic Se could increase Se content up to 46.04 μg·g^-1^ in strawberry (Mimmo et al., 2017) (Lu et al., 2022). Although it has been regarded that daily intake of Se content exceeding 400 μg is harmful for humans (Yao et al., 2022), considering that Se deficiency is more prevalent than its poisoning, most countries, including China, have removed selenium from food pollutant lists. Currently, there is no national or international standard to restrict Se content in foods. In the present study, we increased the Se content up to 29.33 μg ·kg^-1^·FW in strawberry through foliar spraying with 100 mg·L^-1^ Na_2_SO_3_ (**Figure 3**), which is still too low as a Se supplementary food source. It has been generally regarded that under proper agronomic management, foliar application of Se in the early developmental stage could accumulate higher levels of Se in plants (Hu et al., 2022). Thus, the accumulation of Se in strawberry fruit can be promoted by spraying seedlings with Na_2_SO_3_, so as to promote the production of Se-enriched strawberries in the future.

Moreover, numerous studies have reported that Se has been increasingly used to improve the quality of horticultural crops in different forms. For instance, it was reported that glucosamine selenium at a certain concentration greatly increased the content of Se, flavonoids, and mineral elements in tea, along with improving the antioxidant system (Xu et al., 2024). The foliar application of selenomethionine markedly decreased the MDA and H_2_O_2_ content and improved the strawberry fruit quality parameters of soluble solids, soluble sugar, sugar-acid ratio, and AsA, as well as the antioxidant enzyme activities (Wang et al., 2022). There is evidence of an increase in TPC and anthocyanins in plants treated with Se (Liu et al., 2017). In the present study, our results showed that Na_2_SO_3_ treatment largely increased the content of AsA, TPC, anthocyanins, and DPPH, but reduced the MDA content (**Figure 4**), confirming the positive effects of Se on improving the nutritional quality and antioxidant system. However, our results showed that TFC was significantly diminished by Se treatment (**Figure 4C**), which might be due to the downregulation of the expression of genes involved in the flavonoid biosynthesis pathway by Se treatment as mentioned in summer tea (Huang et al., 2023). However, the mechanism underlying the regulation of flavonoids by exogenous Se needs to be further explored. Moreover, our results also showed an inhibition of proanthocyanidin accumulation in Se-treated strawberry fruit (**Figure 4F**), which could be explained by the increase in anthocyanin accumulation and competition of substances because anthocyanin and proanthocyanidin biosynthesis share a common upstream pathway and the same precursors (Dixon et al., 2013).

Sugars and organic acids are the main indicators of strawberry fruit quality and taste. Similar to previous studies (Mimmo et al., 2017; Gao et al., 2022), our results have shown that strawberries with Se-biofortification had significantly higher levels of total soluble sugars but lower TA levels, thus greatly higher sugar-acid ratio (**Figure 5**). However, the potential mechanisms have rarely been explored. To gain insights into the molecular mechanism underlying the sugar and acid regulation by exogenous Se treatment, the changes in the composition and content of the predominant sugars and organic acids and the corresponding enzyme activities, as well as the gene expressions during fruit development and ripening, were investigated in this study. Consequently, it was found that fructose was the most abundant, followed by sucrose and glucose in ripened strawberry fruit (**Figures 6A–C**). As previously reported, fructose and glucose are the main soluble sugars, while sucrose exists at a lower level in strawberries (Milosavljević et al., 2023). The difference in sucrose content in this study might be due to the cultivar differences. In the sugar metabolism pathway, SSs, especially SPS, are mainly responsible for the synthesis of sucrose, while SS-c, AI, and NI promote sucrose hydrolysis to glucose and fructose (Gao et al., 2022). The present study showed that the enzyme activities of SPS, SSc, and AI were upregulated, but SSs and NI were not significantly altered by Se treatment at the end of fruit development (**Figures 7A–E**). Furthermore, there was a significant increase of sucrose, glucose, and fructose (**Figures 6A–C**), suggesting that Se application contributed to both sucrose synthesis and decomposition in strawberries. Correspondingly, the transcript abundance of *FaSPS* was significantly increased by Se treatment (**Figure 7G**), which supported the enzyme activity change (**Figure 7C**). However, the expression of *FaAI* was slightly lower in Se-treated fruit than that in the control fruit (**Figure 7I**), indicating that Se affected AI mainly at the enzyme level rather than the gene expression level. Furthermore, CS and PEPC are key enzymes involved in organic acid biosynthesis, while the increase in IDH activity will promote the degradation of citric acid (Zhang and Fernie, 2018). In the present study, the activities of CS and PEPC were significantly downregulated, while the IDH activity was notably upregulated during fruit development and ripening by Se treatment compared to control (**Figures 8A–C**), which might be the main reason for the increase in organic acid content in strawberries treated with Se (**Figures 6D, E**). Interestingly, the tendency of *FaCS*, *FaPEPC*, and *FaIDH* expression was not consistent with the enzyme activities under Se treatment (**Figures 8D–F**), demonstrating that Se regulated organic acid metabolism mainly by influencing the involved steps at the enzyme activity level, not at the gene expression level. However, here we only focused on the effects of foliar sodium selenite application on particular involved enzymes and gene expression. The utilization of Se in different forms and ways, and more enzymes, metabolites and gene expression need to be integratively analyzed to fully understand the regulatory role of Se in sugar and organic acid metabolism in strawberry.

## 5 Conclusions

This study demonstrated that foliar Se application effectively improved the growth, antioxidant activity, and fruit quality of strawberries. It could increase the contents of Se and several other phytochemicals such as AsA, TPC, and anthocyanins. In particular, Se treatment could increase the ratio of soluble sugars to acid. The content of soluble sugars (sucrose, glucose, and fructose) and organic acid including citric acid and malic acid was respectively upregulated and downregulated during fruit development and ripening, by regulating the activities of the corresponding involved enzymes and transcript levels, providing an insight into the regulatory mechanism of Se in sugar and acid metabolism. In a summary, foliar spraying of Se could be used as an alternative technology to regulate strawberry plant growth and improve fruit quality by increasing Se, AsA, TPC, and anthocyanin content and regulating sugar and acid metabolism.

## Data Availability

The original contributions presented in this study are included in the paper/**Supplementary Material**, and any further inquiries can be directed to the corresponding author.
